# Abortion of Typhoid Fever

**Published:** 1885-10

**Authors:** 


### ABORTION OF TYPHOID FEVER.

AND now comes Edwin R. Maxson, M. D., A. M., LL. D., Syracuse, N. Y. (New York Medical Journal), and claims that he can, and does abort typhoid fever! In the paper referred to he says: '' I will state the means by which I have succeeded in arresting, within one week, nearly every case clearly diagnosticated as typhoid or enteric fever that I have treated during the past ten years.'' He does it thus: '' To destroy the poison in the blood,'' he gives four grains (to adult) sulpho-carbolate of sodium every six hours. ''To sustain the sinking powers,'' he gives two grains cinchonidine in ten drops muriated tincture of iron every six hours. ''To unload the liver'' he gives an improved compound cathartic pill, ''one at first, and one a day if the bowels are confined.'' ''To call the circulation to the extremities and the skin into action,'' a hot foot bath morning and evening. '' But should the headache continue in spite of all this,'' says the Doctor, ''which is rarely the case,'' he takes a teaspoonful of blood, by cups, from the nape of the neck, and repeats it, if necessary, or applies a blister. ''To subdue thoracic, abdominal and other irritations,'' he applies "daily, from the very first, warm sinapisms over the chest and entire adbomen, mornings and evenings." "To keep up the spirits" he lets the patient remain up and dressed, and "out of bed days as far as consistent with safety, being allowed to recline on a lounge or to occupy an easy chair—thus securing better sleep nights."

Well, the Doctor has gotten it down pretty fine, we must say. But it does seem to us, from reading the entire paper (of course we cannot do it justice in the few extracts made here), and from one remark made by the writer, that he takes a very superficial view of fever, and seems to think that it is nothing more than an "accumulation of animal heat—from want of due exhalation from the skin," and that it is only necessary to set the skin to acting, to reduce it! We will venture Dr. Maxson would not succeed so easily in aborting some of the typhoid fever the doctors down this way encounter occasionally. We have no faith in any specific for typhoid fever, nor for any plan for "aborting" it. We were a pupil of the lamented Fenner, and that was his hobby—"abortion of typhoid fever"; but he never succeeded in demonstrating the abortion to our satisfaction.

